# Predicting progression to severe COVID-19 using the PAINT score

**DOI:** 10.1186/s12879-022-07466-4

**Published:** 2022-05-26

**Authors:** Ming Wang, Dongbo Wu, Chang-Hai Liu, Yan Li, Jianghong Hu, Wei Wang, Wei Jiang, Qifan Zhang, Zhixin Huang, Lang Bai, Hong Tang

**Affiliations:** 1 Center of Infectious Diseases, West China Hospital, Sichuan University, 37 Guoxue Lane, Chengdu, Sichuan Province 610041 People’s Republic of China; 2 COVID-19 Medical Team (Hubei) of West China Hospital, Sichuan University, Chengdu, 610041 People’s Republic of China; 3 The People’s Hospital of Qianxi, Qianxi, 551500 People’s Republic of China; 4 The People’s Hospital of Duyun, Duyun, 558000 People’s Republic of China; 5 Emergency Department, West China Hospital, Sichuan University, Chengdu, 610041 People’s Republic of China; 6 Department of Obstetrics and Gynecology, Renmin Hospital of Wuhan University, Wuhan, 430060 People’s Republic of China

**Keywords:** COVID-19, SARS-CoV-2, NK cell, Prediction

## Abstract

**Objectives:**

One of the major challenges in treating patients with coronavirus disease 2019 (COVID-19) is predicting the severity of disease. We aimed to develop a new score for predicting progression from mild/moderate to severe COVID-19.

**Methods:**

A total of 239 hospitalized patients with COVID-19 from two medical centers in China between February 6 and April 6, 2020 were retrospectively included. The prognostic abilities of variables, including clinical data and laboratory findings from the electronic medical records of each hospital, were analysed using the Cox proportional hazards model and Kaplan–Meier methods. A prognostic score was developed to predict progression from mild/moderate to severe COVID-19.

**Results:**

Among the 239 patients, 216 (90.38%) patients had mild/moderate disease, and 23 (9.62%) progressed to severe disease. After adjusting for multiple confounding factors, pulmonary disease, age > 75, IgM, CD16^+^/CD56^+^ NK cells and aspartate aminotransferase were independent predictors of progression to severe COVID-19. Based on these five factors, a new predictive score (the ‘PAINT score’) was established and showed a high predictive value (C-index = 0.91, 0.902 ± 0.021, p < 0.001). The PAINT score was validated using a nomogram, bootstrap analysis, calibration curves, decision curves and clinical impact curves, all of which confirmed its high predictive value.

**Conclusions:**

The PAINT score for progression from mild/moderate to severe COVID-19 may be helpful in identifying patients at high risk of progression.

**Supplementary Information:**

The online version contains supplementary material available at 10.1186/s12879-022-07466-4.

## Introduction

In December 2019, a cluster of pneumonia cases of unknown origin was reported in Wuhan [1, 2]. The pathogen of the novel pneumonia was identified to be a novel β-coronavirus, currently named severe acute respiratory syndrome coronavirus 2 (SARS-CoV-2), and has close phylogenetic similarity to SARS-CoV [3]. SARS-CoV-2 infection has been named coronavirus disease 2019 (COVID-19) by the World Health Organization (WHO) [4].

COVID-19 has now become a worldwide health concern. The estimated overall case-fatality rate for COVID-19 is approximately 1–2.3%, which is similar to that of Spanish influenza (2–3%) and much higher than that of seasonal influenza (0.1%) [5–7]. The severity of COVID-19 has been classified as mild, moderate, severe and critical per the WHO-China Joint Mission [8]. Among a total of 72 314 case records from the Chinese Center for Disease Control and Prevention, approximately 81% of COVID-19 cases were defined as mild, 14% of COVID-19 cases were severe, and 5% were critical [5]. The overall hospital mortality of COVID-19 cases is approximately 15–20%, but it is up to 40–49% among critical cases requiring intensive care unit admission [2, 5]. Therefore, it is important to evaluate the risk factors for disease progression in COVID-19 patients. Early identification of COVID-19 patients with possible progression of the disease is particularly important for optimal treatment choice and reducing mortality.

Studies have revealed some changes in haematologic and immunologic tests and have investigated risk factors for mortality and outcomes in patients with COVID-19 [2, 7, 9–13]. Older age, high Sequential Organ Failure Assessment (SOFA) score, d-dimer greater than 1 µg/mL, lymphocytopenia and history of coronary vascular disease were reported to increase the risk of death in patients with COVID-19 [7, 9–11].

Moreover, score prediction models, such as prognostic nutritional index (PNI), Systemic immune-inflammatory index (SII) have been used to predict prognosis in COVID-19 patients and other diseases [14–18]. However, the risk factors related to the progression of COVID-19 symptoms from mild/moderate to severe are still limited and need to be assessed. The immune response and inflammatory cytokines are important to analyse to elucidate the mechanisms of host responses and pathogenesis of COVID-19 [10, 19–21]. In addition to decreased T cells, natural killer (NK) cell immunotypes were recently reported to be related to COVID-19 disease severity [19, 22]. We enrolled patients with COVID-19 from two medical centres in China and aimed to evaluate the risk factors for progression to severe-stage disease based on the new combined score including NK cell information.

## Materials and methods

### Study design and participants

This study was a retrospective, multicentre study. Laboratory-confirmed COVID-19 admitted cases from Renmin Hospital of Wuhan University and West China Hospital of Sichuan University were reviewed. We included adult patients (aged ≥ 18 years) admitted between February 6 and April 6, 2020, with SARS-CoV-2 infection confirmed by RT-PCR. Informed consent was exempted because it was reported as grouped data with no identifying factors.

The diagnosis of COVID-19 was made according to the WHO interim guidance [23]. The severity of COVID-19 was assessed according to the Seventh Version of the Novel Coronavirus Pneumonia Diagnosis and Treatment Guidance from the National Health Commission of China [24]. According to the guidelines, patients were categorized into the mild, moderate, severe, or critical group upon admission [24].

### Data collection

The clinical data, laboratory data and radiological data of all COVID-19 patients were obtained from the electronic medical records of the treating hospital. Data were reviewed and verified by a team of physicians (Dongbo Wu, Wei Jiang, Changhai Liu, Ming Wang and Lang Bai). Any missing or unclear records were collated and clarified through communication with local medical staff or patients and their families.

Detailed demographic information, comorbidities, symptoms, and disease severity of all patients were recorded or diagnosed on admission. Clinical and virological characteristics were recorded, e.g., age, sex, past medical history, and clinical findings, e.g., white blood cell (WBC) count, neutrophil count (NEU), lymphocyte count (LYM), haemoglobin (HGB), platelet count (PLT), prothrombin time (PT), D-dimer, alanine aminotransferase (ALT), aspartate aminotransferase (AST), γ-glutamyl transpeptidase (GGT), albumin (ALB), total bilirubin (TBIL), direct bilirubin (DBIL), uric acid (UA), creatinine (Cr), creatine kinase (CK), lactate dehydrogenase (LDH), brain natriuretic peptide (BNP), procalcitonin (PCT), c-reactive protein (CRP), neutrophils/lymphocyte ratio (NLR), other biochemical parameters and SARS-CoV2 RNA test results. There were no cases lost to follow-up in this study.

### Statistical analysis

The baseline clinical characteristics and outcomes of the patients were assessed. Categorical data are presented as frequencies (percentages); continuous variables are presented as medians (range, minimum–maximum). The demographic, clinical and laboratory characteristics of COVID-19 patients were compared among groups. The Mann–Whitney U test was used to compare continuous variables. The χ^2^ test with Yates’ correction was used for 2 × 2 contingency data, and Pearson’s χ^2^ test was used for contingency data for variables with more than two categories.

To explore risk factors, or their interactions, associated with COVID-19 severity, univariable and multivariable logistic regression models were used to estimate odds ratios (ORs) and 95% confidence intervals (CIs). A Cox proportional hazards model was constructed sequentially introduced variables, and a significance level of p > 0.05 was used to remove variables from the model. Final model selection was performed by backward selection of all factors. Schoenfeld and Martingale residuals were used to check the proportional hazards assumption and nonlinearity, respectively.

Survival curves were compared using the Kaplan–Meier method (log-rank test) since time-to-mortality and time-to-event are crucial in interpreting the results. Estimates of adjusted hazard ratios (HRs), 95% CIs, and p values are displayed. Harrell’s concordance index (C-index) was used to assess the score’s discrimination ability. C-index values and the corresponding 95% CIs were estimated for each main study time point. In addition, bootstrapping, calibration curves, decision curves and clinical impact curves were applied to verify the nomogram. A two-sided p < 0.05 was considered statistically significant. All statistical analyses were performed using R software (version 3.5.2, http://CRAN.R-project.org, R Foundation, Vienna, Austria).

### Ethics approval and consent to participate

This study was approved by the Ethics Committee of West China Hospital of Sichuan University (NO. 2020-444) and was allowed exemption from the requirement of informed consent. All research was conducted in accordance with the Declaration of Helsinki. Raw data generated or analysed during this study are provided in Additional file 8. 

## Results

### Demographic and clinical characteristics of the study population

A total of 239 COVID-19 patients were included in this study. In the present cohort, 216 (90.38%) patients had mild/moderate disease, and 23 (9.62%) patients experienced progression to severe disease. The demographic and clinical characteristics of the study population are presented in Table 1. The median age was 58 years (range 26–90 years), and 58.20% (139/239) were male. The median maximum temperature was 38 °C (range 36.0–41.0).

**Table 1:** Demographic and clinical characteristics of the study population

Characteristic	All patients (n = 239)	Without progression (n = 216)	Progression to severe (n = 23)	p value
Sex Male n (%)	139 (58.2)	130 (60.2)	9 (39.1)	0.038
Age (year)	58 (26–90)	57.5 (26–88)	62 (29–90)	0.010
Max temperature (℃)	38.0 (36.0–41.0)	38.0 (36.0–39.8)	38.3 (36.5–41.0)	0.129
Clinical manifestations, n (%)				
Fever	186 (77.8)	168 (77.8)	18 (78.3)	0.688
Cough	144 (60.3)	127 (58.8)	17 (73.9)	0.175
Expectoration	57 (23.8)	50 (23.1)	7 (30.4)	0.642
Dyspnea	25 (10.5)	23 (10.7)	2 (8.7)	0.001
Chest pain	11 (4.6)	10 (4.6)	1 (4.3)	0.982
Angina	10 (4.2)	8 (3.7)	2 (8.7)	0.662
Fatigue	68 (28.5)	58 (26.9)	10 (43.5)	0.206
Myalgia	22 (9.2)	22 (10.2)	0	0.221
Headache	12 (5.0)	12 (5.6)	0	0.476
Vomit	4 (1.7)	3 (1.4)	1 (4.3)	0.421
Diarrhea	43 (18.0)	40 (18.5)	3 (13.0)	0.787
Comorbidities, n (%)				
Hypertension	66 (27.6)	60 (27.8)	6 (26.1)	0.782
Diabetes mellitus	25 (10.5)	20 (9.3)	5 (21.7)	0.285
Cardiac disease	20 (8.4)	19 (8.8)	1 (4.3)	0.509
Pulmonary disease	10 (4.2)	7 (3.2)	3 (13.0)	0.035
Liver disease	11 (4.6)	8 (3.7)	3 (13.0)	0.027

When comparing demographic data at admission, patients with progression to severe disease were more likely to be male and older aged (> 75 years) than patients without progression (*p* < 0.05, Table 1). The clinical manifestations were mainly as follows (Table 1): fever 77.8% (186/239), cough 60.3% (144/239), expectoration 23.8% (57/239), dyspnoea 10.5% (25/239), chest pain 4.6% (11/239), angina 4.2% (11/239), fatigue 28.5% (68/239), myalgia 9.2% (22/239), headache 5.0% (12/239), vomiting 1.7% (4/239) and diarrhoea 18% (43/239).

The clinical characteristics of the study population are summarized in Table 2. When comparing biochemical indexes of COVID-19 between moderate cases with and without progression, we found that there were significant differences in lymphocytes, NLR, CRP, AST, TBIL, DBIL, Cr, urea, glucose, sodium, PT, CD3^+^ T cells, CD4^+^ T cells, CD8^+^ T cells, CD19^+^ T cells, CD16^+^/ CD56^+^ NK cells between patients with and without progression (all *p* < 0.05); detailed information are listed in Table 2.

**Table 2:** The lab test and clinical characteristics of the study population

Laboratory findings	All patients (n = 239)	Without progression (n = 216)	Progression to severe (n = 23)	p value
Systolic pressure (mmHg)	129.00 (87–195)	128 (87–195)	133 (108–151)	0.775
Diastolic pressure (mmHg)	78 (51–114)	78 (51–106)	75 (60–114)	0.131
Rhythm of the heart (beats/min)	85 (37–140)	85 (37–140)	86 (75–114)	0.796
Breathing rate (beats/min)	20 (15–32)	20 (15–25)	20 (16–32)	0.008
CURB-65 score	0 (0–3)	0 (0–2)	0 (0–3)	0.497
qSOFA score	0 (0–3)	0 (0–2)	0 (0–3)	0.035
WBC (× 10^9^/L)	5.07 (1.23–17.48)	5.06 (1.23–17.48)	5.27 (2.55–11.38)	0.529
Neutrophils (× 10^9^/L)	3.08 (0.69–16.18)	3.03 (0.69–16.18)	3.75 (1.16–9.53)	0.672
Percentage of neutrophils (%)	63.5 (23.1–97.7)	62.0 (23.1–97.7)	68.9 (35.9–94.3)	0.000
Lymphocyte (× 10^9^/L)	1.22(0.29–3.48)	1.29 (0.31–3.48)	0.94 (0.29–1.64)	0.001
NLR	2.41 (0.42–32.21)	2.28 (0.42–27.90)	3.47 (0.72–32.21)	0.019
Percentage of lymphocyte (%)	25.9 (2.9–59.2)	26.6 (3.3–59.2)	20.7 (2.9–50.2)	0.000
HGB (g/L)	125 (65–159)	125 (65–159)	119 (90–149)	0.545
PLT (× 10^9^/L)	217 (27–608)	219.5 (27–608)	210 (128–490)	0.078
CRP (mg/L)	38.1 (3–181)	35.9 (3–181)	64.7 (6–171)	0.058
ALT (IU/L)	22 (6–274)	21 (6–274)	28 (9–162)	0.171
AST (IU/L)	22(10–312)	21 (10–312)	30 (13–104)	0.017
GGT (IU/L)	24 (0.85–3-8)	23 (0.85–309)	28 (10–105)	0.084
TBIL (µmol/L)	10.0 (2.5–36.8)	10.1 (2.5–36.8)	9.7 (6.0–19.1)	0.010
DBIL (µmol/L)	3.5 (0.6–13.6)	3.5 (0.6–13.6)	4.1 (2.6–9.2)	0.020
Urea (mmol/L)	4.3(1.7–39.2)	4.16 (1.70–39.2)	5 (2.91–28.1)	0.010
Cr (µmol/L)	58 (35–1045)	58 (35–1045)	65 (37–231)	0.017
UA (µmol/L)	267 (69–683)	267 (69–683)	274 (106–536)	0.293
Glucose (mmol/L)	5.37 (3.06–34.2)	5.36 (3.06–34.20)	5.55 (3.12–23.04)	0.001
Potassium (µmol/L)	3.97 (2.52–7.97)	3.96 (2.52–7.97)	4.07 (3.44–5.84)	0.402
Sodium (mmol/L)	142 (126–154)	142 (126–154)	139 (131–148)	0.004
Calcium (mmol/L)	2.16 (1.53–4.35)	2.17 (1.53–4.35)	2.03 (1.89–4.35)	0.525
Magnesium (mmol/L)	0.84 (0.21–2.07)	0.84 (0.21–1.13)	0.87 (0.63–2.07)	0.926
Cholesterol (mmol/L)	3.96 (2.14–9.97)	4.00 (2.29–9.97)	3.38 (2.14–4.58)	0.960
Triacylglycerol (mmol/L)	1.24 (0.28–8.40)	1.24 (0.28–8.40)	1.26 (0.49–2.53)	0.178
PT (s)	11.7 (10.2–17.9)	11.7 (10.2–17.9)	12.4 (11–14.3)	0.029
Fibrinogen (g/L)	3.73 (1.05–19.7)	3.61 (1.05–19.7)	4.62 (2.08–6.78)	0.001
D-dimer (mg/L)	0.515 (0.100–55.3)	0.47 (0.10–55.3)	0.92 (0.29–18.07)	0.420
CK (IU/L)	0.84 (0.18–9.51)	0.82 (0.18–9.51)	1.16 (0.34–2.46)	0.875
Myoglobin (ng/mL)	32.38 (9.92–282.83)	31.44 (9.92–282.83)	40.78 (18.15–270.34)	0.004
BNP (pg/mL)	67.44 (0–5398.00)	59.82 (0–5398)	121.65 (6.72–1150.00)	0.728
PCT (ng/L)	0.042 (0–4.320)	0.04 (0–4.32)	0.065 (0–1.080)	0.000
CD3^+^ T cell (/μL)	807 (164–2284)	825 (164–2284)	510 (166–1182)	0.002
CD4^+^ T cell (/μL)	484(59–1705)	508 (59–1705)	378 (59–754)	0.003
CD8^+^ T cell (/μL)	262(43–951)	271.5 (43–951)	195 (79–417)	0.008
CD19^+^ T cell (/μL)	152 (18–986)	156 (18–986)	123 (31–395)	0.031
CD16^+^/CD56^+^ NK cell (/μL)	128 (12–677)	134 (12–677)	88 (22–278)	0.012
Ig A (g/L)	2.22 (1–11)	2.22 (1–11)	2.36 (1–8)	0.074
Ig G (g/L)	11.4 (6–30)	11.3 (6–30)	12.6 (6–17)	0.947
Ig M (g/L)	0.89 (0.26–2.52)	0.92 (0.26–2.52)	0.70 (0–2)	0.040
Ig E (g/L)	37.7 (0–2220)	38 (0–2220)	33.6 (0–590)	0.897

Quick sequential organ failure assessment, qSOFA; White blood cell, WBC; neutrophil count, NEU; Neutrophils/Lymphocyte ratio, NLR; hemoglobin, HGB; platelet count, PLT; prothrombin time, PT; alanine aminotransferase, ALT; aspartate aminotransferase, AST; γ-glutamyl transpeptidase, GGT; albumin, ALB; total bilirubin, TBIL; Direct bilirubin, DBIL; uric acid, UA, creatinine, Cr; creatine kinase, CK; lactate dehydrogenase, LDH; Brain Natriuretic Peptide, BNP; procalcitonin, PCT; c-reactive protein, CRP

### Univariant and multivariant Cox regression model for progression from mild/moderate to severe disease

**Fig. 1:**
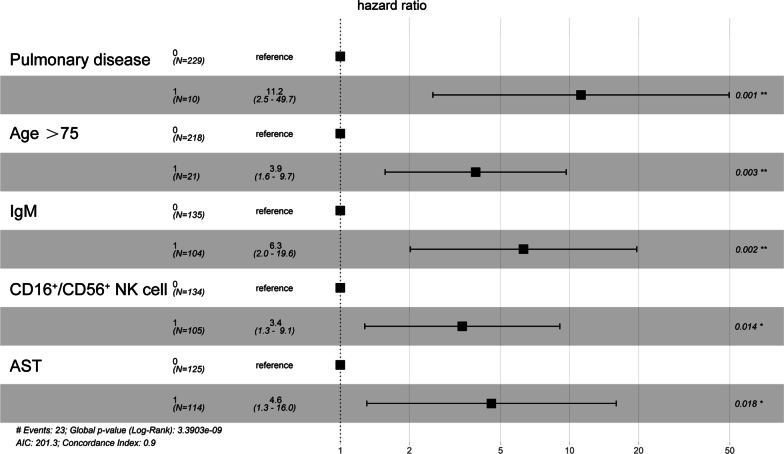
Forest plot of significant factors in the Cox proportional hazards regression model. Shown in the figure are the HR and the 95% CI associated with the end point

When exploring risk factors for progression from mild/moderate to severe COVID-19, we compared the demographic and clinical data of moderate cases and cases with progression to severe disease. Using univariant and multivariant Cox regression models, the results showed a significant difference in pulmonary disease (11.20, 95% CI 2.50–49.70, p = 0.001), age over 75 (3.92, 95% CI 1.61–9.73, p = 0.003), IgM (6.31, 95% CI 1.99–19.60, p = 0.002), CD16^+^/CD56^+^ NK cells (3.40, 95% CI 1.31–9.13, p = 0.014) and AST (4.60, 95% CI 1.31–16.00, p = 0.018) (Table 3), which were the 5 independent risk factors for progression from mild/moderate to severe disease (Fig. 1). However, there were no significant impacts by other variables in our study population (see Additional file 6: Table S1). We also used the global Schoenfeld test of Cox diagnostic deviance and Cox proportional hazards model fit to evaluate these five independent risk factors for progression from mild/moderate to severe disease, which suggested good performance (see Additional file 1: Fig. S1, Additional file 2: Fig. S2, Additional file 3: Fig. S3).

**Table 3:** Univariant and multivariant COX regression model for progression from mild/moderate cases into severe case

Variables	Univariable logistic regression		Multivariable logistic regression	
	OR (95%)	p value	OR (95%)	p value
General information				
Age, > 75 years	1.03 (1.01–1.06)	0.01	3.92 (1.61–9.73)	0.003
Sex, male	1.494 (1.02–2.184)	0.038	1.67 (0.55–5.09)	0.364
Comorbidities				
Pulmonary disease	3.625 (1.092–12.032)	0.035	11.20 (2.50–49.70)	0.001
Liver disease	3.304 (1.146–9.527)	0.027	1.27 (0.26–6.34)	0.768
Laboratory findings				
Lymphocyte < 1 × 10^9^/L	0.215 (0.087–0.529)	0.001	0.73 (0.19–2.81)	0.646
NLR	1.074 (1.012–1.140)	0.019	0.73 (0.19–2.81)	0.646
AST > 40 U/L	1.008 (1.001–1.020)	0.017	4.60 (1.31–16.00)	0.018
Urea (mmol/L)	1.066 (1.020–1.115)	0.005	1.92 (0.64–5.71)	0.243
PT (s)	1.482 (1.042–2.109)	0.029	1.63 (0.63–4.24)	0.315
Cr > 133 mol/L	1.008 (1.001–1.015)	0.017	2.87 (1.18–6.98)	0.02
CD4^+^ T cell (/μL)	0.097 (0.995–0.999)	0.003	0.5 (0.05–5.12)	0.556
CD8^+^ T cell (/μL)	0.996 (0.992–0.999)	0.008	1.18 (0.25–5.52)	0.830
CD3^+^ T cell (/μL)	0.998 (0.997–0.999)	0.002	2.32 (0.2–27.42)	0.505
CD19^+^ T cell (/μL)	0.995 (0.990–0.999)	0.031	1.13 (0.29–4.41)	0.861
CD16^+^/CD56^+^ NK cell (/μL)	0.992 (0.985–0.998)	0.012	3.40 (1.31–9.13)	0.014
Ig M (g/L)	0.260 (0.072–0.941)	0.040	6.31 (1.99–19.60)	0.002

*NLR* Neutrophils/Lymphocyte ratio, *PT* prothrombin time, *AST* aspartate aminotransferase, *Cr* creatinine

Moreover, the Kaplan–Meier survival curve analysis and log-rank test showed a significant difference in survival curve in COVID-19 patients categorized by pulmonary disease, age, IgM, CD16 + /CD56 + NK cells and AST (see Additional file 4: Fig. S4a–e).

### Development of a predictive score for progression from moderate to severe disease

Predictors including pulmonary disease, age, IgM, CD16 + /CD56 + NK cells and AST were enrolled in the development of predictive scores for COVID-19 patient progression from mild/moderate to severe disease. The new predictive score (pulmonary disease, age, **I**gM, CD16^+^/CD56^+^ **N**K cell, AS**T**; PAINT score) = (pulmonary disease) × 2.4174 + (age > 75) × 1.3594 + (IgM < 0.84) × 1.8399 + (CD16^+^/CD56^+^ NK cell < 116.5) × 1.2246 + (AST > 25) × 1.5182.

**Fig. 2:**
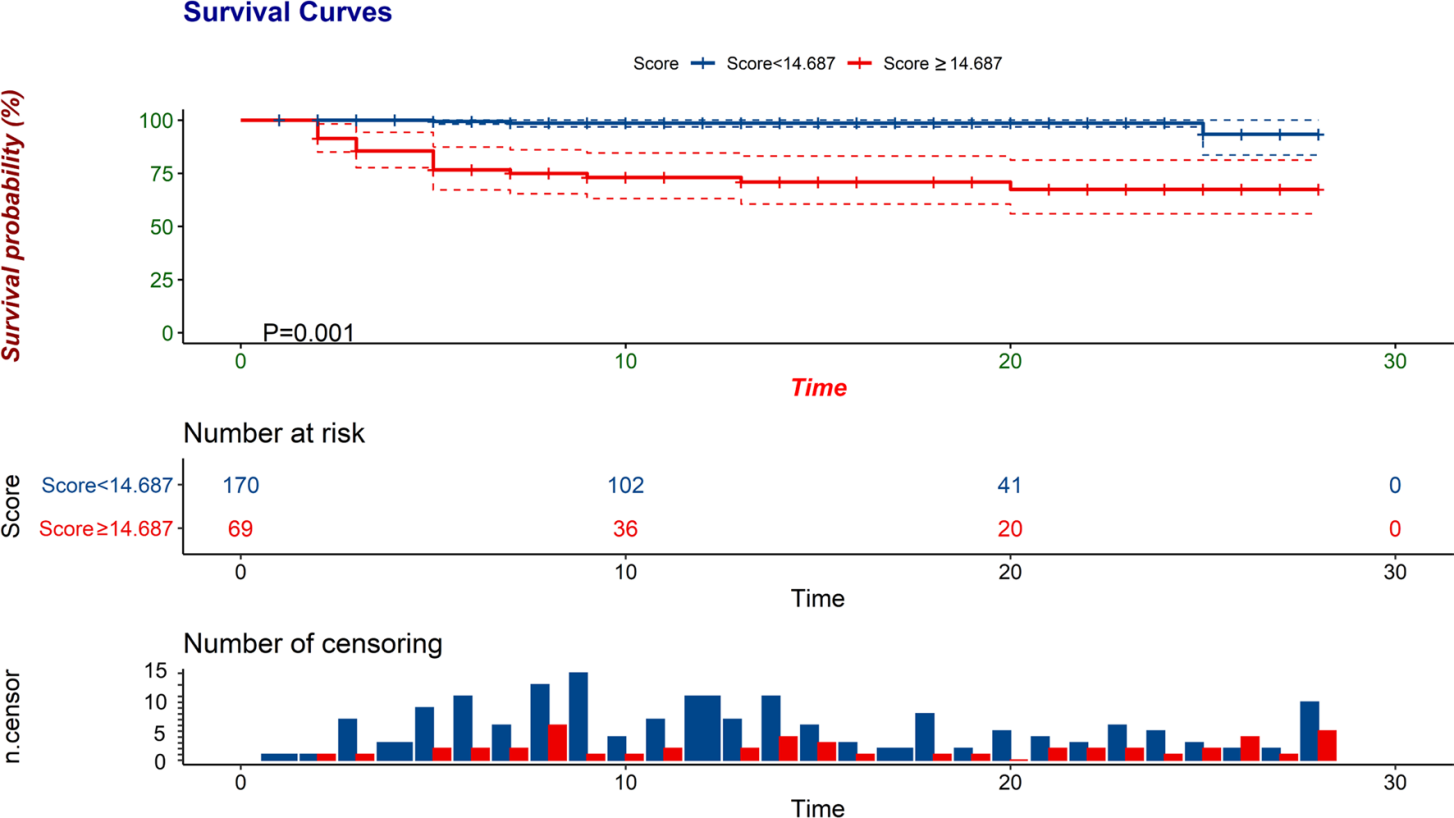
Kaplan–Meier survival curve analysis of the PAINT score

The points contributing to each variable are shown in Additional file 5: Fig. S5. To demonstrate the ability of the new predictive score to identify more severe patients for early clinical treatment, Kaplan–Meier survival curve analysis was used to find the best cut-off value; a value of 14.687 points was found to divide the patients into mild/moderate and progression to severe disease groups (P = 0.001, Fig. 2).

**Fig. 3:**
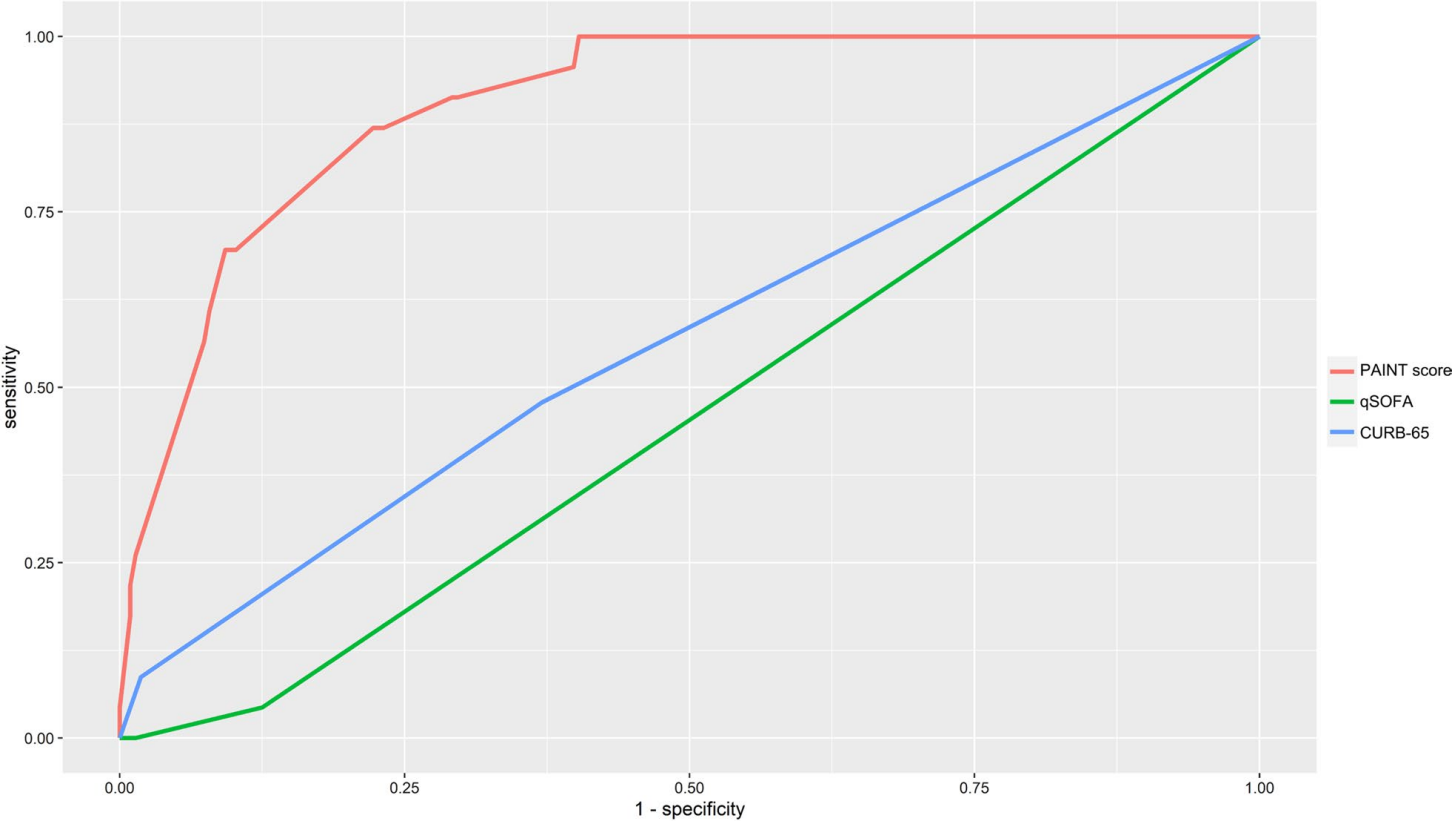
ROC analysis was used to evaluate the efficacy of the PAINT score model for predicting COVID-19 patients’ progression from mild/moderate to severe disease. C-index values and the corresponding 95% CIs were estimated for each of the main study time points to assess the score’s discrimination ability. P values represent the statistical significance of the differences between the new score and the other prognostic score or factor

We performed ROC analysis to evaluate the efficacy of the PAINT score model for predicting COVID-19 patients’ progression from mild/moderate to severe disease. We compared the PAINT score with the qSOFA and CURB-65 (confusion, uraemia, respiratory rate, BP, age > 65 years) scores. As demonstrated in Fig. 3, the C-index of the new predictive progression model for predicting progression from mild/moderate to severe disease was 0.902 ± 0.021. However, the C-index of the qSOFA and CURB-65 scores for the prediction of progression was 0.534 ± 0.027 and 0.561 ± 0.058, respectively. We also compared the new predictive progression model with the 5 independent risk factors (pulmonary disease, age, IgM, CD16 + /CD56 + NK cells and AST), and the C-index for the prediction of progression was 0.5432 ± 0.034, 0.639 ± 0.052, 0.683 ± 0.044, 0.647 ± 0.050 and 0.716 ± 0.036, respectively (see Additional file 7: Table S2). Moreover, we evaluated the predictive value of the PNI and SII score in our study population, the C-index was 0.814 ± 0.042 and 0.769 ± 0.039, respectively (see Additional file 7: Table S2). These findings suggested that the PAINT score might be suitable for predicting progression from mild/moderate to severe disease.

**Fig. 4:**
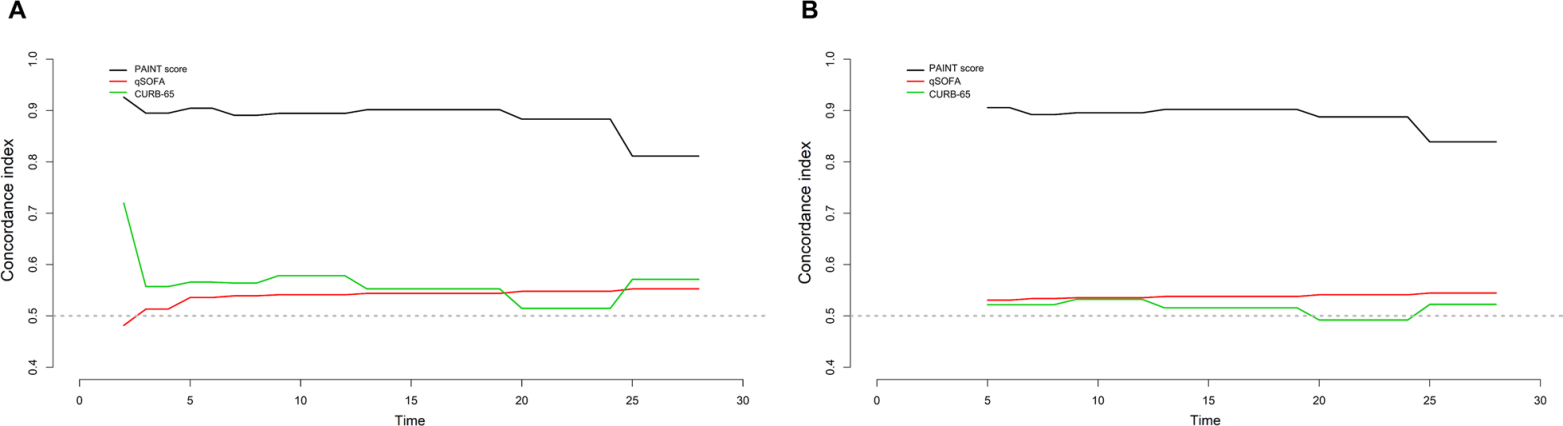
For internal validation of the discriminability of the PAINT score model, we performed concordance index analysis (**A**) and 1000 bootstrap replicates (**B**)

For internal validation of the ability of the new predictive progression model, we performed concordance index analysis to evaluate the discrimination of the PAINT score. Better discrimination was observed with our PAINT score than with the qSOFA and CURB-65 scores (Fig. 4a). Moreover, we performed 1000 bootstrap internal validations, and our new predictive PAINT score also showed better discrimination (Fig. 4b).

### Nomogram, calibration, decision curve and clinical impact curve for progression from mild/moderate to severe disease

**Fig. 5:**
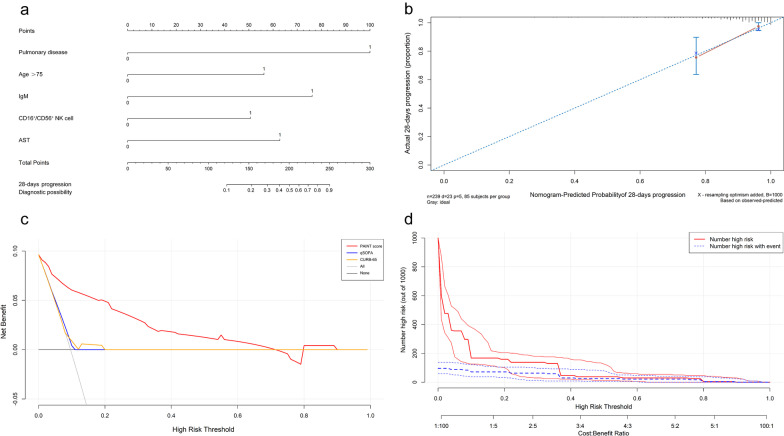
Nomogram, calibration curve, decision curves and clinical impact curves for progression from mild/moderate to severe disease. **a** Nomogram. To use the nomogram, the value of an individual patient is located on each variable axis, and a line is drawn upward to determine the number of points received for each variable value. The sum of these numbers is located on the total point axis, and a line is drawn downward to the survival axes to determine the likelihood of 28-day progression to severe disease. **b** Calibration. The nomogram-predicted probability of nonsevere survival is plotted on the x-axis, and that of actual nonsevere survival is plotted on the y-axis. **c** Decision curve. The abscissa of this graph is the threshold probability, and the ordinate is the net benefit. **d** Clinical impact curve. The red curve (number of high-risk individuals) indicates the number of people who are classified as positive (high risk) by the model at each threshold probability; the blue curve (number of high-risk individuals with outcome) is the number of true positives at each threshold probability

In our study population, we used 5 variables (pulmonary disease, age, IgM, CD16 + /CD56 + NK cells and AST) to predict 28-day progression from mild/moderate to severe disease. According to the principles of nomogram score construction, each variable is given different points and weights. The nomogram score is shown in Fig. 5a. We evaluated the score of each variable in turn according to its clinical characteristics and examination results and then summarized the score according to the total score of the 5 variables. Based on the total score, the probability of progression to severe COVID-19 can be determined. The calibration curves for 28-day progression were also well defined in the internal validation set (Fig. 5b). Nomogram and decision curve analyses also indicated good performance of the PAINT score (Fig. 5c). Clinical impact curves were used to assess the clinical usefulness of the risk prediction nomogram (Fig. 5d).

## Discussion

By using univariant and multivariant Cox regression models, we identified five independent risk factors (pulmonary disease, age, IgM, CD16^+^/CD56^+^ NK cell and AST) for progression to severe COVID-19 in the present study. We developed a new predictive score, the PAINT score, for progression to severe disease and found that a value of 14.687 points divided the patients into mild/moderate and progression to severe disease groups. We also established a new nomogram score to predict 28-day progression from mild/moderate to severe disease. These results may be important to predict progression of moderate COVID-19 to severe disease and may be helpful in identifying cases of potential progression in a timely manner to improve the prognosis.

SARS-CoV-2 is a single-stranded RNA virus that infects cells through its structural spike (S) protein binding the angiotensin-converting enzyme 2 (ACE2) receptor [25]. Then, the type 2 transmembrane serine protease (TMPRSS2) receptor cleaves ACE2, activating the S protein, which promotes virus uptake and mediates SARS-CoV-2 entry into host cells [2, 25]. Both ACE2 and TMPRSS2 are expressed in host cells, particularly the alveolar epithelial type II cells of COVID-19 patients [20]. COVID-19 has various clinical manifestations, and the common symptoms in hospitalized patients include fever (70–90%), dry cough (60–86%), shortness of breath (53–80%), fatigue (38%), myalgias (15–44%), nausea/vomiting or diarrhoea (15–39%), headache, weakness (25%), and rhinorrhoea (7%) [2]. In a retrospective study of 548 patients with COVID-19 in China, most patients with severe/critical and fatal disease presented with sputum and dyspnoea much more often than those with mild/moderate disease on admission who survived [4]. Eighty-one percent of patients had mild manifestations, 14% had severe manifestations, and 5% had critical manifestations (defined as respiratory failure, septic shock, and/or multiple organ dysfunction). A study of 20,133 hospitalized patients in the UK reported that the most common major comorbidities were chronic cardiac disease (30.9%), diabetes (20.7%), chronic pulmonary disease excluding asthma (17.7%), and chronic kidney disease (16.2%) [26]. In our study population, the most common major comorbidity was pulmonary disease. Moreover, we found that pulmonary disease was an independent risk factor for progression to severe COVID-19. The limited sample size may be responsible for this difference with the previous UK report [26].

We used Cox regression methods to explore the risk factors related to progression to severe COVID-19. Risk factors related to progression were reported in nonsevere COVID-19 patients, such as lymphocyte count, neutrophil count, CD4^+^ and CD8^+^ T cell counts, CRP, D-dimer, interleukin-6, interleukin-8, lactate dehydrogenase, age, dyspnoea on admission, and hypertension [10, 27–29]. From the present study, there were significant differences in lymphocytes, NLR, CRP, AST, Cr, CD3^+^ T cells, CD4^+^ T cells, CD8^+^ T cells, CD19^+^ T cells, and CD16^+^/CD56^+^ NK cells in mild/moderate COVID-19 cases with and without progression. We also found that pulmonary comorbidities had an impact on the risk of progression. In another study, four variables (comorbidity, dyspnoea on admission, lactate dehydrogenase and lymphocyte count) were included in a predictive model. A total score of 6 points was used to divide patients into high-risk and low-risk groups [27]. Moreover, the risk factors for progression to severe illness in COVID-19 patients with cancer not only included the previous variables of older age, interleukin 6, procalcitonin, D-dimer, and lymphocytes but also included tumour stage, tumour necrosis factor α, N-terminal pro-B-type natriuretic peptide, CD4^+^ T cells and albumin–globulin ratio [12, 13, 30]. Many reports have linked diabetes and obesity to more severe COVID-19 illness and worth progress [31]. In our study cohort, 10.5% patients had diabetes mellitus, including 9.3% in patients without progression and 21.7% in patients with progression. However, there was no significant difference of diabetes in patients with and without progression. This may be because of our sample size. It is necessary to further expand the sample size in the future study.

There were many predict score models for disease severity in COVID-19 patients, including early warning score, National Early Warning Score 2, q-COVID score, prognostic nutritional index score, Brescia‑COVID Respiratory Severity Scale score, systemic immune-inflammatory index score, COVID-GRAM score, etc. [18, 32–34] However, the important information of immune cells was not involved. To predict the risk of progression, we developed the new, predictive PAINT score which contained the NK cells. Using a value of 14.687 points, we could divide the patients into mild/moderate and progression to severe disease groups, with a higher C-index (0.902 ± 0.021) than that obtained with the qSOFA (0.534 ± 0.027), CURB-65 scores (0.561 ± 0.058), PNI score (0.814 ± 0.042) and SII score (0.769 ± 0.039). The internal validation of discrimination and 1000 bootstrap replicates showed the good ability of our new predictive progression model. Moreover, other risk models for COVID-19 death or mortality were reported and evaluated, such as APACHE II, SIRS, SOFA, qSOFA, COVID-19 score, COVID-PIRO score, the COVID-19 Risk of Complications Score, Pneumonia Severity Index, etc. [35–38] The Specification and validation of COVID-19 scoring systems should be performed and verified in the large real-world cohort study in the future.

The immune response to SARS-CoV-2 is key for the control and resolution of COVID-19 infection. T cells also play important roles in the immune response to SARS-CoV-2 infection. Lymphocytopenia was found to be one of the most common features in laboratory tests of COVID-19 patients, and reduced CD4^+^ and CD8^+^ T cell counts were predictive of disease progression [10, 11]. In addition to decreased levels of CD3^+^/CD4^+^ T lymphocytes, CD3^+^/CD8^+^ T lymphocytes and CD19^+^ B lymphocytes, CD16^+^/CD56^+^ NK cells were also decreased in the peripheral blood of COVID-19 patients, and these cells may play critical roles in the inflammatory cytokine storm [21]. NK immunotypes are related to COVID-19 disease severity, and high expression of perforin, NKG2C, and Ksp37 in NK cells may reflect the increased presence of adaptive NK cells in the circulation of patients with severe disease [19]. This may be the mechanism of NK cell activation in COVID-19 and the potential role of NK cells in host protection and immunopathology [19]. Compared with mild cases, significantly lower levels of immune cells including CD3 + T cell, CD4 + T cell, CD8 + T cell, B cell and NK cell were found in severe cases [39–42]. In our cohort, CD3^+^ T cells, CD4^+^ T cells, CD8^+^ T cells, CD19^+^ T cells, and CD16^+^/ CD56^+^ NK cells showed significant differences in mild/moderate COVID-19 cases with and without progression. Moreover, CD16^+^/CD56^+^ NK cells were also independent risk factors for progression from moderate to severe disease. In a multi-center study, Benjamin Kramer et al. [42] reported that the dysfunction of NK cell not only affects antiviral immune responses but may also be related to the development of fibrotic lung disease in severe COVID-19 cases. From the pathologic mechanism, untimely early production of TGFβ and associated NK cell dysfunction is a hallmark of severe COVID-19 [43]. TGFβ-mediated impairment of NK cell function may reduce virus control and be detrimental in severe COVID-19 cases [43]. A detailed map of the NK cell activation landscape in COVID-19 disease might be a meaningful indicator of progression.

Our study has several limitations. First, the study population only included patients from Renmin Hospital of Wuhan University (Central China region) and West China Hospital of Sichuan University (Southwest China region). The study sample size was relatively small, it must still be considered preliminary information. We plan to apply the PAINT score to further validate the predict value in the future studies. Second, the data were obtained from the electronic medical database of the two hospitals. Some cases had incomplete records for the exposure history and laboratory examinations, and some patients were diagnosed in the outpatient department, with incomplete medical records and laboratory testing that was only briefly documented. Third, many patients remained in the hospital, and the outcomes were unknown at the time of data collection. Fourth, detailed follow-up information was not included in our study results. Therefore, the uncertainty of bias might have inevitably affected our assessment. Further evaluation may be needed to validate our predictive model.

## Conclusion

In conclusion, pulmonary disease, age, IgM, CD16 + /CD56 + NK cells and AST were independent predictors of progression for patients with COVID-19 in the present study. A predictive model for progression to severe COVID-19 based on the PAINT score might be helpful to identify patients at risk of progression. Moreover, more intensive surveillance and appropriate therapy should be considered in patients at high risk of progression to improve their prognosis in clinical practice. Future studies with larger numbers of patients will be useful for updating and validating this PAINT score to improve identification of patients at risk of progression to severe COVID-19.

## Supplementary Information

**Additional file 1: Figure S1.** Global Schoenfeld test cox diagnostics deviance to evaluate five independent risk factors for progression from mild/moderate into sever cases. Schoenfeld residual were displays in graphs. (a) Pulmonary disease, (b) Age, (c) IgM, (d) CD16^+^/CD56^+^ NK cell, (e) AST.

**Additional file 2: Figure S2.** Dfbeta were displayed by diagnostics graphs presenting goodness of Cox Proportional Hazards Model fit.

**Additional file 3: Figure S3.** Deviance residuals were displayed by diagnostics graphs presenting goodness of Cox Proportional Hazards Model fit. (a) pulmonary disease, (b) Age, (c) IgM, (d) CD16^+^/CD56^+^ NK cell, (e) AST.

**Additional file 4: Figure S4.** The Kaplan–Meier survival curve analysis and log-rank test of each independent predictors showed a significant difference in survival curve in COVID-19 patients. (a) Pulmonary disease, (b) Age, (c) IgM, (d) CD16^+^/CD56^+^ NK cell, (e) AST.

**Additional file 5: Figure S5.** The Principal Component Analysis (PCA) showed the configuration of biomarkers on biplot represented the relationship between variables and principal (a, b).

**Additional file 6: Table S1.** Univariant logistic regression model for progression from mild/moderate cases into severe cases.

**Additional file 7: Table S2.** The C-index for the prediction of progression for predicting progression from mild/moderate cases into severe cases.

**Additional file 8.** Supplmentary data raw data.

## Data Availability

Additional file 8 is included. All data generated or analysed during this study are included in this published article and its additional information files.
