# Supplementary figures and images for: Predicting progression to severe COVID-19 using the PAINT score
Source: BMC Infect Dis. 2022 May 26;22:498. doi: 10.1186/s12879-022-07466-4 (PMC9134988; doi:10.1186/s12879-022-07466-4)

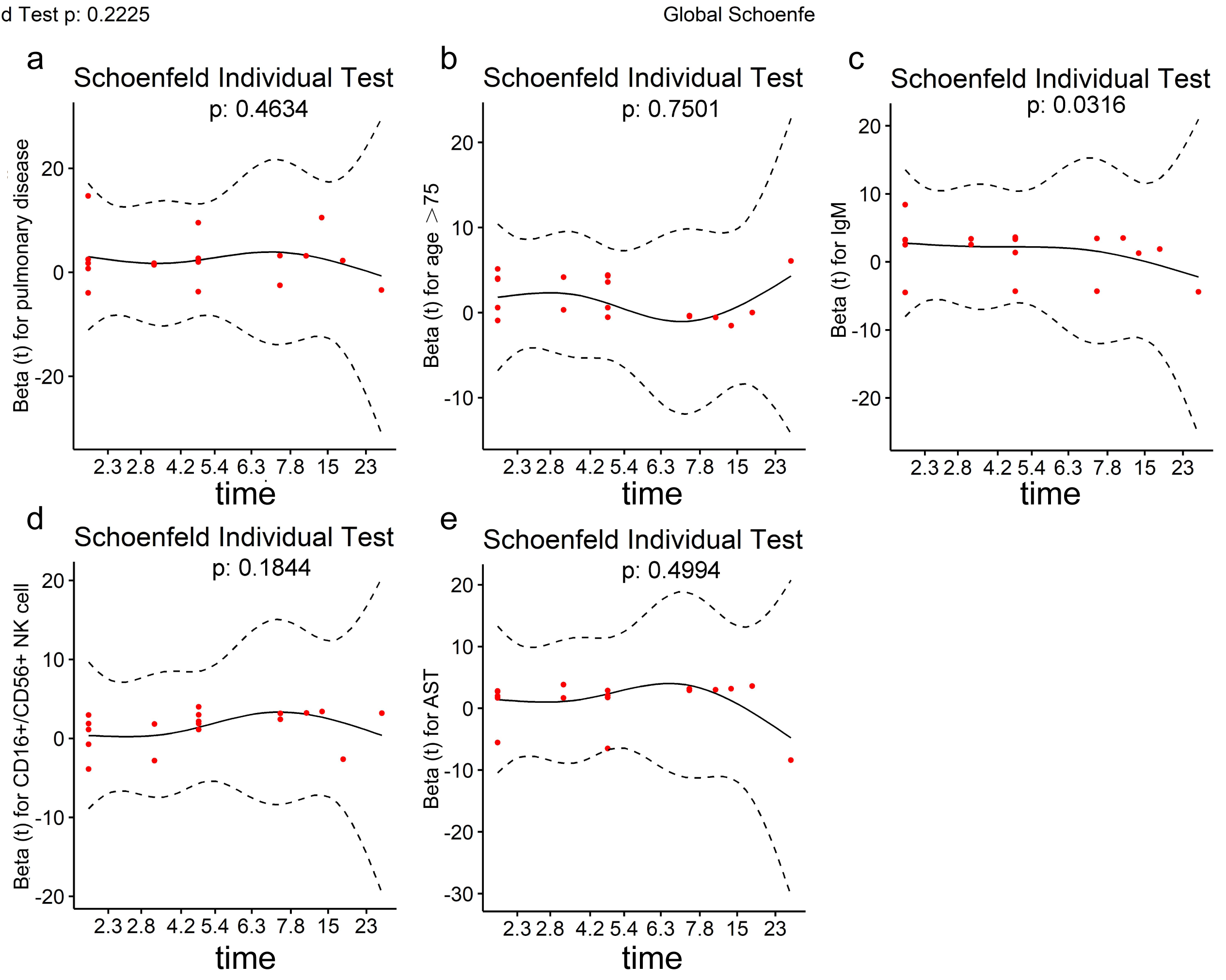

Supplement: Supplementary file 1 — Additional file 1: Figure S1. Global Schoenfeld test cox diagnostics deviance to evaluate five independent risk factors for progression from mild/moderate into sever cases. Schoenfeld residual were displays in graphs. (a) Pulmonary disease, (b) Age, (c) IgM, (d) CD16+/CD56+ NK cell, (e) AST. [file 12879_2022_7466_MOESM1_ESM.tif]

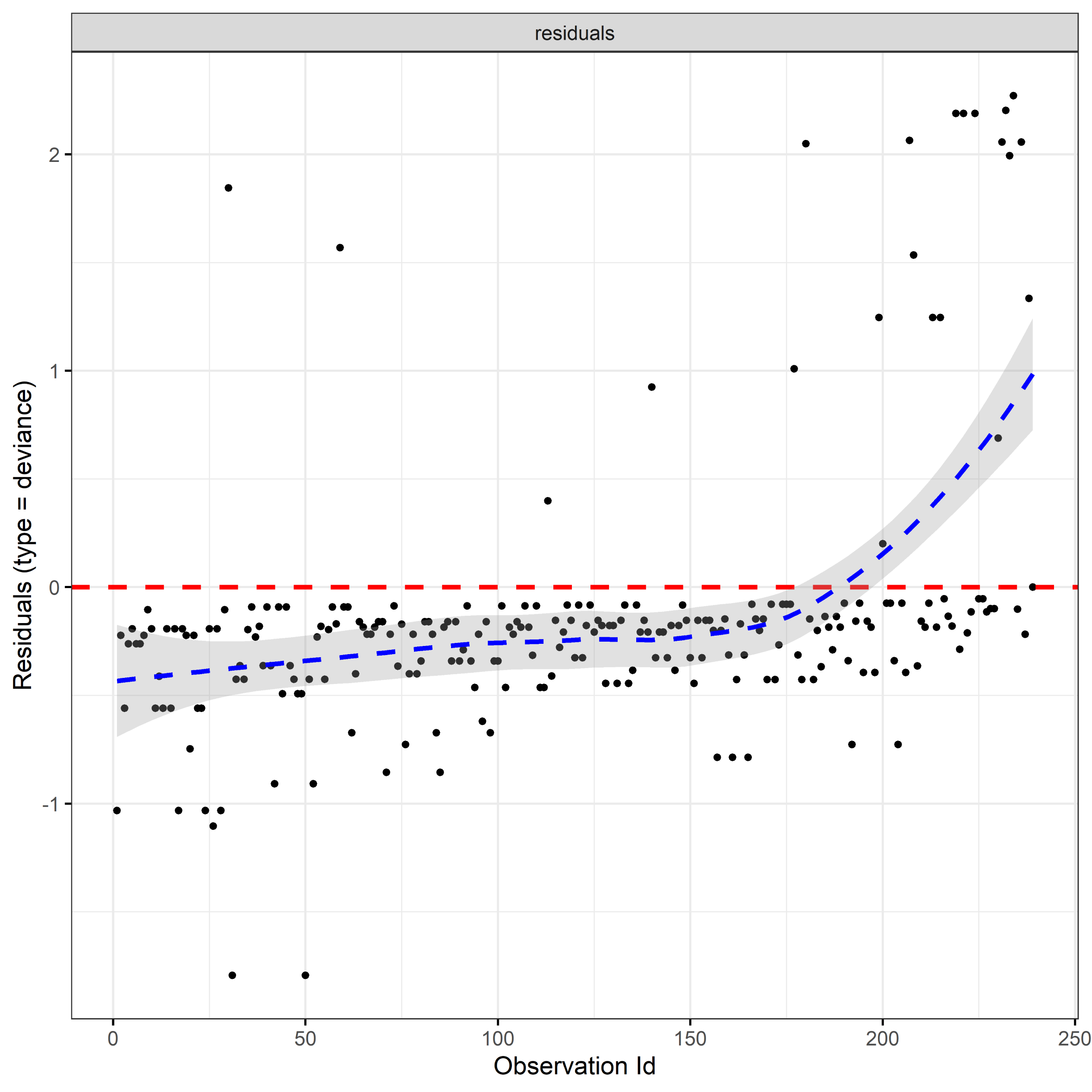

Supplement: Supplementary file 2 — Additional file 2: Figure S2. Dfbeta were displayed by diagnostics graphs presenting goodness of Cox Proportional Hazards Model fit. [file 12879_2022_7466_MOESM2_ESM.tif]

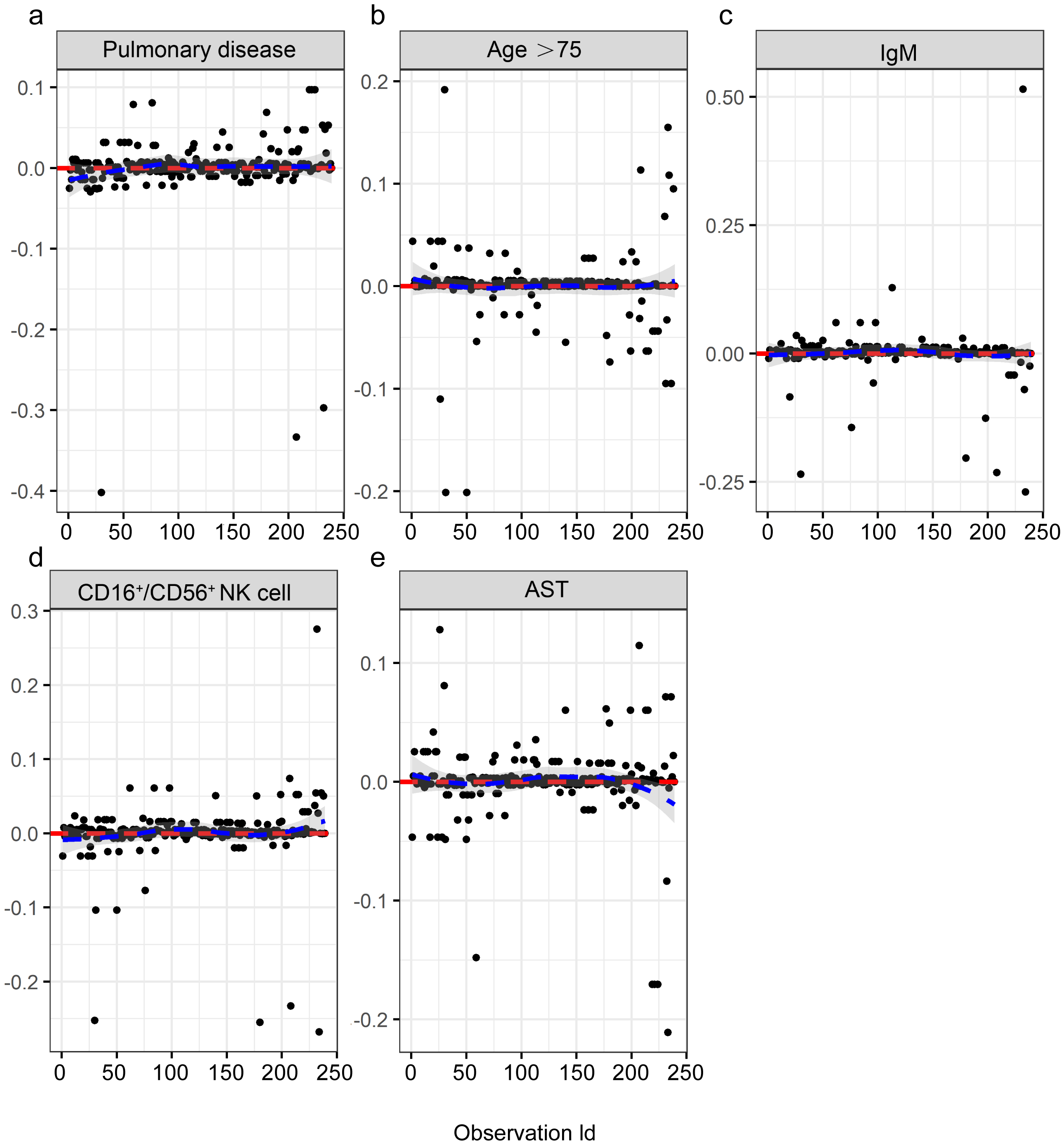

Supplement: Supplementary file 3 — Additional file 3: Figure S3. Deviance residuals were displayed by diagnostics graphs presenting goodness of Cox Proportional Hazards Model fit. (a) pulmonary disease, (b) Age, (c) IgM, (d) CD16+/CD56+ NK cell, (e) AST. [file 12879_2022_7466_MOESM3_ESM.tif]

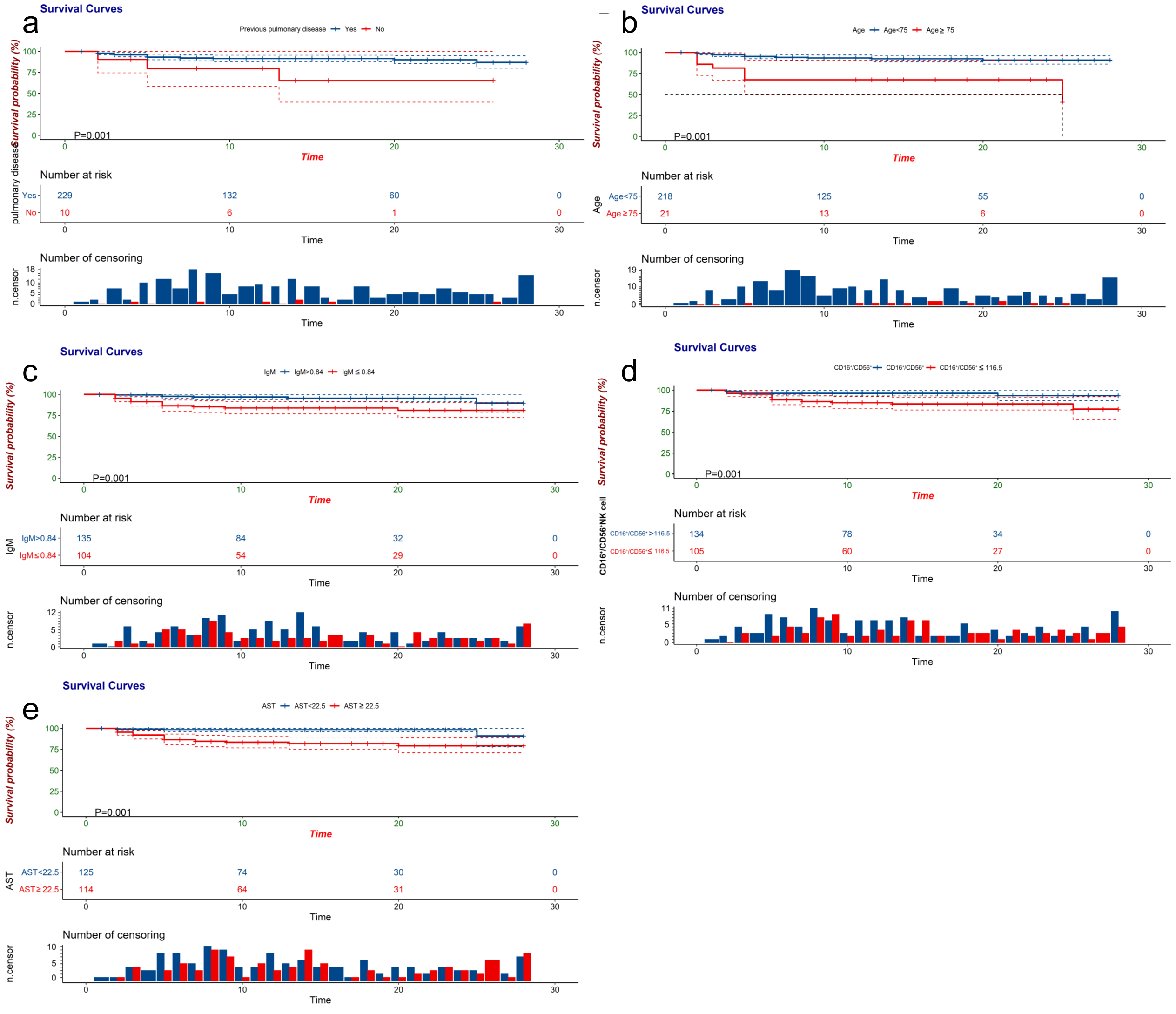

Supplement: Supplementary file 4 — Additional file 4: Figure S4. The Kaplan–Meier survival curve analysis and log-rank test of each independent predictors showed a significant difference in survival curve in COVID-19 patients. (a) Pulmonary disease, (b) Age, (c) IgM, (d) CD16+/CD56+ NK cell, (e) AST. [file 12879_2022_7466_MOESM4_ESM.tif]

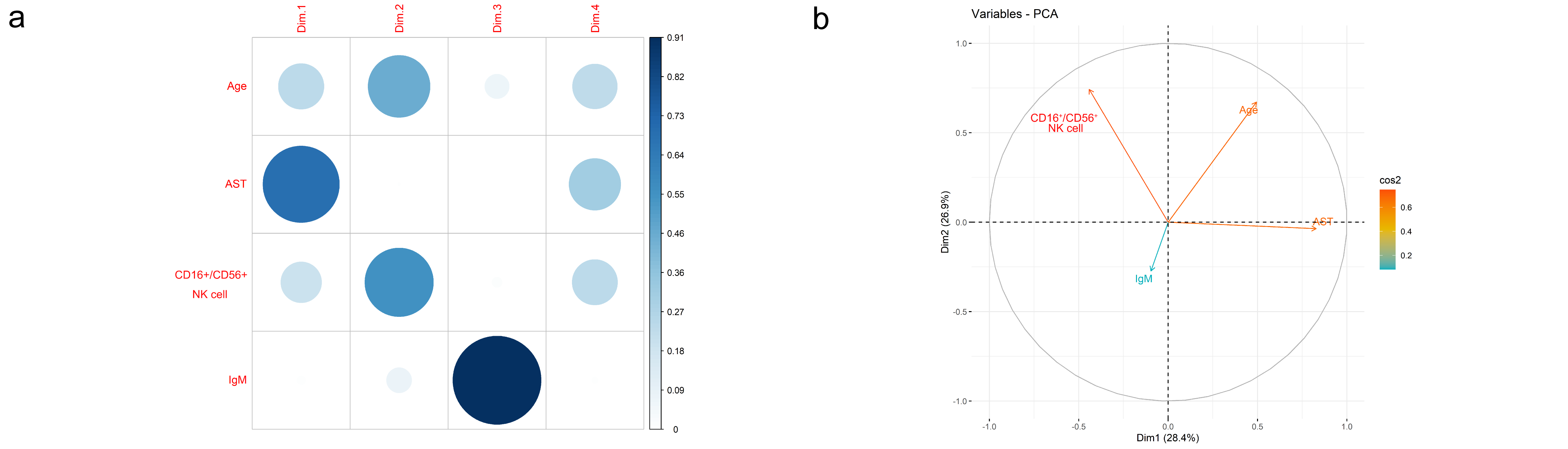

Supplement: Supplementary file 5 — Additional file 5: Figure S5. The Principal Component Analysis (PCA) showed the configuration of biomarkers on biplot represented the relationship between variables and principal (a, b). [file 12879_2022_7466_MOESM5_ESM.tif]
