# Supplementary material for: Predicting progression to severe COVID-19 using the PAINT score
Source: BMC Infect Dis. 2022 May 26;22:498. doi: 10.1186/s12879-022-07466-4 (PMC9134988; doi:10.1186/s12879-022-07466-4)
Supplement: Supplementary file 6 — Additional file 6: Table S1. Univariant logistic regression model for progression from mild/moderate cases into severe cases. [file 12879_2022_7466_MOESM6_ESM.docx]

**Table S1 Univariant logistic regression model for progression from mild/moderate cases into severe cases.**

| Variables | Univariable logistic regression | |
| --- | --- | --- |
|  | OR (95%) | P value |
| Comorbidities | | |
| Hypertension | 0.880 (0.356-2.173) | 0.782 |
| Diabetes | 1.696 (0.643-4.474) | 0.285 |
| Cardio diseases | 0.510 (0.069-3.757) | 0.509 |
| Tumor | 1.958 (0.463-8.285) | 0.362 |
| Clinical manifestations | | |
| Fever | 1.204 (0.487-2.980) | 0.688 |
| Cough | 1.809 (0.768-4.259) | 0.175 |
| Expectoration | 1.215 (0.535-2.762) | 0.642 |
| Chest pain | 0.983 (0.134-7.237) | 0.987 |
| Angina | 1.379 (0.326-5.822) | 0.662 |
| Fatigue | 1.631 (0.764-3.484) | 0.206 |
| Myalgia | 0.326 (0.044-2.398) | 0.271 |
| Headache | 0.047 (0.000-215.360) | 0.476 |
| Vomit | 2.087 (0.282-15.444) | 0.471 |
| Diarrhea | 0.864 (0.300-2.494) | 0.787 |
| Laboratory findings | | |
| WBC >10×10^9^/L | 0.949 (0.807-1.116) | 0.529 |
| NEU (x10^9^/L) >0.50 vs ≤0.50 | 1.033 (0.889-1.200) | 0.672 |
| lymphocytes (x10^9^/L) >0.35 vs ≤0.35 | 0.215 (0.087-0.529) | 0.001 |
| PLT<150×10^9^/L | 0.996 (0.991-1.000) | 0.078 |
| HGB <110 g/L | 0.994 (0.975-1.103) | 0.545 |
| CRP >10 mg/L | 1.010 (1.001-1.020) | 0.058 |
| ALT>40 U/L | 1.006 (0.998-1.014) | 0.171 |
| GGT (IU/L) | 1.006 (0.999-1.013) | 0.084 |
| UA (µmol/L) | 1.002 (0.998-1.005) | 0.293 |
| BNP (pg/mL) | 1.000 (0.999-1.000) | 0.728 |
| D-dimer (mg/L) | 1.021 (0.970-1.075) | 0.420 |
| CK (IU/L) | 1.034 (0.686-1.558) | 0.875 |
| CD4^+^/ CD8^+^T cell ratio | 0.021 (0.719-1.450) | 0.909 |
| Ig A (g/L) | 1.235 (0.980-1.557) | 0.074 |
| Ig E (g/L) | 1.000 (0.998-1.002) | 0.897 |
| Ig G (g/L) | 1.004 (0.894-1.128) | 0.947 |
| C3 (g/L) | 0.732 (0.087-6.174) | 0.775 |
| C4 (g/L) | 0.832 (0.014-48.701) | 0.929 |

Note: White blood cell, WBC; neutrophil count, NEU; hemoglobin, HGB; platelet count, PLT; prothrombin time, ALT; aspartate aminotransferase, GGT; uric acid, UA, creatine kinase, CK; Brain Natriuretic Peptide, BNP.
