# Supplementary material for: Predicting progression to severe COVID-19 using the PAINT score
Source: BMC Infect Dis. 2022 May 26;22:498. doi: 10.1186/s12879-022-07466-4 (PMC9134988; doi:10.1186/s12879-022-07466-4)
Supplement: Supplementary file 7 — Additional file 7: Table S2. The C-index for the prediction of progression for predicting progression from mild/moderate cases into severe cases. [file 12879_2022_7466_MOESM7_ESM.docx]

**Table S2 The C-index for the prediction of progression for predicting progression from mild/moderate cases into severe cases.**

|  | ROC comparison | |
| --- | --- | --- |
|  | C-Index | P value |
| PAINT score | 0.902 ± 0.021 |  |
| qSOFA score | 0.534 ± 0.027 | p<0.001 |
| CURB-65 score | 0.561 ± 0.058 | p<0.001 |
| Prognostic nutritional index (PNI) | 0.814 ± 0.042 | p<0.001 |
| Systemic immune-inflammatory index (SII) | 0.769 ± 0.039 | p<0.001 |
| Pulmonary disease | 0.543 ± 0.034 | p<0.001 |
| Age | 0.639 ± 0.052 | p<0.001 |
| IgM | 0.683 ± 0.044 | p<0.001 |
| CD16^+^/ CD56^+^ NK cell | 0.647 ± 0.050 | p<0.001 |
| AST | 0.716 ± 0.036 | p<0.001 |

NOTE: Quick sequential organ failure assessment, qSOFA; aspartate aminotransferase, AST.
